# Sex Differences in Gray Matter Volume of the Right Anterior Hippocampus Explain Sex Differences in Three-Dimensional Mental Rotation

**DOI:** 10.3389/fnhum.2016.00580

**Published:** 2016-11-15

**Authors:** Wei Wei, Chuansheng Chen, Qi Dong, Xinlin Zhou

**Affiliations:** ^1^Advanced Technology Innovation Center for Future Education, Beijing Normal UniversityBeijing, China; ^2^Department of Psychology and Behavioral Sciences, Zhejiang UniversityHangzhou, China; ^3^State Key Laboratory of Cognitive Neuroscience and Learning, Beijing Normal UniversityBeijing, China; ^4^Department of Psychology and Social Behavior, University of CaliforniaIrvine, CA, USA

**Keywords:** sex difference, mental rotation, anterior hippocampus, brain structure

## Abstract

Behavioral studies have reported that males perform better than females in 3-dimensional (3D) mental rotation. Given the important role of the hippocampus in spatial processing, the present study investigated whether structural differences in the hippocampus could explain the sex difference in 3D mental rotation. Results showed that after controlling for brain size, males had a larger anterior hippocampus, whereas females had a larger posterior hippocampus. Gray matter volume (GMV) of the right anterior hippocampus was significantly correlated with 3D mental rotation score. After controlling GMV of the right anterior hippocampus, sex difference in 3D mental rotation was no longer significant. These results suggest that the structural difference between males’ and females’ right anterior hippocampus was a neurobiological substrate for the sex difference in 3D mental rotation.

## Introduction

The hippocampus plays an important role in spatial processing. The “place cells” in the hippocampus are the core part in the “GPS” of the brain that processes spatial information (O’Keefe, 1976; Fyhn et al., 2004; Sargolini et al., 2006). Both animal and human studies have shown that the hippocampal volume is related to spatial ability. For example, homing pigeons have larger hippocampi than non-homing pigeons (Rehkämper et al., 1988). The birds that need to store their food have larger hippocampi than the ones who do not (Krebs et al., 1989; Sherry et al., 1989). In terms of the mammals, the rats that store their food in a distributed manner have larger hippocampi than the ones that store their food in one central location (Jacobs and Spencer, 1994).

Human studies also showed that the hippocampus plays an important role in spatial processing (for a recent summary see Lee et al., 2012). First, patients who suffered from developmental topographical disorders (DTD) have damages to the hippocampus (in addition to the retrosplenial cortex, fusiform gyrus, and lingual gyrus) and show impaired performance on navigation tasks (Aguirre and D’Esposito, 1999; Iaria and Barton, 2010). Damages to the hippocampus also result in a deficit in spatial working memory (Abrahams et al., 1997; Bohbot et al., 1998; Holdstock et al., 2000). Second, neuroimaging studies found that the hippocampus is activated by navigation tasks (Maguire et al., 1998; Mellet et al., 2000; Hartley et al., 2003; Iaria et al., 2003, 2008) and spatial memory tasks (Maguire, 1997; Burgess et al., 2002). Moreover, when performing the spatial memory task, only spatial strategies (but not non-spatial strategies) elicited activation in the hippocampus (Iaria et al., 2003; Bohbot et al., 2004). Third, the size of the hippocampus may change dynamically due to a long-term or sustained demand of spatial processing. Experienced taxi drivers have larger posterior hippocampi than the control subjects (Maguire et al., 2000). Spatial navigation training has been found to increase the gray matter of the hippocampus (Lövdén et al., 2010, 2012; Kühn and Gallinat, 2014; Kühn et al., 2014).

Neuroimaging studies have further shown differentiated functions of the anterior and posterior hippocampus (Chua et al., 2007; Ghetti et al., 2010; DeMaster and Ghetti, 2013). Based on the results of 54 previous studies, Lepage et al. (1998) proposed the Hippocampus Encoding/Retrieval (HIPER) Model that specifies the anterior hippocampus’s role in acquiring or encoding new visuospatial information and the posterior hippocampus’s role in retrieval. Subsequent studies have further supported this model (Strange and Dolan, 2001; Hartley et al., 2003; Kumaran and Maguire, 2005; Maguire et al., 2006a; Iaria et al., 2007). Most of the previous studies had focused on spatial memory and have thus implicated the posterior hippocampus for such a function. For example, the “place cells” were found only in the posterior hippocampus in monkeys (Colombo et al., 1998). Taxi drivers’ constant retrieval of city maps can lead to increased gray matter volume (GMV) of the posterior hippocampus (Woollett and Maguire, 2011).

If the hippocampus plays a crucial role in visuospatial processing (in particular, the anterior hippocampus’s role in spatial information encoding), would its structural variations explain one of the most well-documented sex differences—that in visuospatial processing? Decades of behavioral research have shown that sex differences in visuospatial ability are stable across different age groups (Voyer et al., 1995) and that males’ advantage is particularly obvious in mental rotation (Linn and Petersen, 1985; Maeda and Yoon, 2013; Reilly and Neumann, 2013; Lütke and Lange-Küttner, 2015). Moreover, compared to 2-dimensional (2D) mental rotation, 3-dimensional (3D) mental rotation (which requires more spatial processing) has a larger and more consistent sex effect across age groups (Peters et al., 1995; Roberts and Bell, 2003; for a meta-analysis, see Linn and Petersen, 1985). Overall, sex differences are robust based on large-scale studies (e.g., 109,612 men and 88,509 women in Maylor et al., 2007, study; 90,000 women and 111,000 men in Lippa et al., 2010 study) and meta-analysis studies (Geiser et al., 2008; Maeda and Yoon, 2013; Reilly and Neumann, 2013). These sex differences are so robust that only special experimental designs (e.g., positive instructions to women, different delivery modes) or training would reduce or sometimes eliminate them (Goldstein et al., 1990; Peters et al., 1995; Roberts and Bell, 2003; Peters, 2005; Feng et al., 2007; Monahan et al., 2008; Moè, 2009; Glück and Fabrizii, 2010; Tzuriel and Egozi, 2010; Maeda and Yoon, 2013).

In support of a possible neural basis of sex differences in mental rotation, especially, 3D mental rotation, neuroimaging studies have found structural and functional differences between male and female hippocampi. Structurally, the total volume of the hippocampus was larger for females than for males (Giedd et al., 1997; Goldstein et al., 2001). Males showed a significant correlation between the size of the hippocampus and age (from 18 years to 42 years), which was not significant for females (Pruessner et al., 2001; Suzuki et al., 2005), suggesting that the growth of the hippocampus occurred earlier in females (Giedd et al., 1997). Functionally, when performing a navigation task, males have been found to depend more on the hippocampus, whereas females depend more on the parietal and frontal lobes (Grön et al., 2000). Thus far, however, no study has examined whether structural differences in the hippocampus would account for sex differences in 3D mental rotation.

In the current study, we examined the relationship between GMV of the hippocampus (especially its anterior part) and sex differences in 3D mental rotation. The anterior part is particularly relevant because mental rotation tasks mainly involve the encoding of spatial information, rather than the retrieval of previously stored spatial information. We hypothesized that males would have larger volumes in the anterior hippocampus than would females, and such a difference would explain a large part of sex differences in 3D mental rotation.

## Materials and Methods

### Participants

Participants were 431 college students (192 males and 239 females, mean age = 19.9 years, ranging from 18 to 24 years) from Beijing Normal University. No participants had a history of neurological or psychiatric disorders or head injury. This study was approved by the Institutional Review Board of the Imaging Center for Brain Research in the Institute of Cognitive Neuroscience and Learning at Beijing Normal University.

### MRI Acquisition

Whole brain structural MRI scans were performed on a Siemens 3T Trio scanner (Munich, Germany) by using a T1-weighted three-dimensional gradient echo sequence (TR = 2350 ms, TE = 3.39 ms; flip angle = 7°; field of view = 100 mm; matrix = 256 × 256; voxel size = 1 mm × 1 mm × 1 mm).

### Behavioral Tasks

The three-dimensional mental rotation task was based on Shepard’s mental rotation task (Shepard and Metzler, 1971; Peters et al., 1995). Participants were presented one 3D picture as the target and four as answer choices, and they were asked to select the answer that matched the target after rotation. This task had 24 items, which were divided into two 3-min blocks. The total score was analyzed.

Motor-Free Visual Perception Test, Third Edition (MVPT-3) was used to test the general visual perception ability (Colarusso and Hammill, 1972). Five categories of visual perception were measured: spatial relationship, visual closure, visual discrimination, visual memory and figure ground. Total score was analyzed. This task was used to control the general visual perception ability.

Raven’s Advanced Progressive Matrices (RAPM; Raven and Court, 1998) was used to assess general intelligence. Subjects were given 30 min to complete as many items as possible. For each test item, subjects were asked to select from several alternatives of the missing segment that would complete a larger pattern. The number of correct trials was analyzed.

### Data Analysis

#### Structural Whole Brain Analysis

Data were analyzed by using voxel-based morphometry (VBM) implemented in Statistical Parametric Mapping (SPM5, Wellcome Department of Cognitive Neurology) and executed in MATLAB (R2012b, Mathworks, Sherborn, MA, USA). Following the procedures described by Ashburner and Friston (2000) and Good et al. (2001), we conducted the following steps of data pre-processing: extraction of the brain, spatial normalization into the stereotactic space by using the standard SPM gray template, segmentation into gray and white matter and CSF compartments, and correction for volume changes induced by spatial normalization (modulation). The spatially normalized images were written in voxels of 1 mm × 1 mm × 1 mm. The smoothing with a 12 mm full width at half maximum (FWHM) isotropic Gaussian kernel was applied. In the current study, the GMV was analyzed. Two-sample *t*-test was used to investigate sex differences in GMV of the hippocampus.

#### Structural ROI Analysis

The current study focused only on the hippocampus arch, which can be divided into three parts: anterior, middle and posterior hippocampus. This segmentation method has been commonly used to study the different parts of hippocampus arch (Huang et al., 2013; Travis et al., 2014; for a review see Malykhin and Coupland, 2015). The segmentation process was executed in MATLAB (Mathworks, Sherborn, MA, USA). A sagittal view of the hippocampus from Anatomical Automatic Labeling (AAL) was projected onto a plane and the maximum values in the right-left direction and the top-down direction were calculated. After marking the hippocampus into four quarters, it was divided into the anterior, middle, and posterior parts, with the ratios of 1:2:1 (Duvernoy, 2005), which were then used as the mask for structural ROI analysis. To control for sex differences in overall brain size, the whole brain GMV was used as a covariate. A separate analysis was conducted without controlling for the whole brain GMV (see Supplementary Material).

Statistical analysis of the behavioral data was conducted in SPSS (SPSS Inc., Chicago, IL, USA).

## Results

### Behavioral Results

The behavioral tasks showed that males performed better than females on the 3D mental rotation task (*F*_(1,429)_ = 5.95, *p* = 0.015, *d* = 0.234). This sex difference became even slightly greater (*F*_(1,427)_ = 7.65, *p* = 0.006, *d* = 0.254), after controlling for general visual perception and intelligence (RAPM) even though these two correlates did not show significant sex differences (for visual perception, *F*_(1,429)_ = 2.77, *p* = 0.097; and for RAPM, *F*_(1,429)_ = 2.43, *p* = 0.120; see Table 1).

**Table 1:** **Mean scores (and standard deviations) and sex differences for the three behavioral tasks**.

Task	Measure	Mean (SD)		*F*
		Male	Female	Sex
3-D mental rotation	Number of correct items in 6 min	20.34 (8.92)	18.42 (7.43)	5.95*
MVTP	Total score	42.79 (4.37)	43.49 (4.24)	2.77
Raven’s advanced progressive matrices	Number of correct items in 30 min	36.65 (4.67)	37.28 (3.71)	2.43

Note: *p < 0.05.

### Structural ROI Analysis

The structural ROI analysis involved six brain areas (bilateral anterior, middle and posterior hippocampus), so we used Bonferroni correction to adjust for the significance thresholds: *p* = 0.05/*6* = 0.008. Controlling for the whole brain GMV, the GMV of the anterior hippocampus was significantly larger for males than for females (*F*_(1,428)_ = 14.92, *p* < 0.008, for the left hemisphere, and *F*_(1,428)_ = 13.44, *p* < 0.008, for the right hemisphere), whereas the posterior hippocampus was significantly larger for females than males (*F*_(1,428)_ = 24.32, *p* < 0.008, for the left hemisphere, and *F*_(1,428)_ = 9.59, *p* < 0.008, for the right hemisphere; see Table 2 and Supplementary Table 1S). There was no significant sex difference in GMV of the middle hippocampus (*F*_(1,428)_ = 0.001, *n.s*., for the left hemisphere; *F*_(1,428)_ = 0.01, *n.s*., for the right hemisphere). After controlling for the whole brain GMV, there also were no significant sex differences in GMV of the whole left hippocampus (*F*_(1,428)_ = 0.07, *n.s*.) and the whole right hippocampus (*F*_(1,428)_ = 0.19, *n.s*.).

**Table 2:** **Mean scores (and standard error) and sex differences in gray matter volumes (mm^3^) of the left and right anterior, middle and posterior hippocampus (with the gray matter volume of the whole brain as a covariate)**.

	Sex		*F*	*p*
	Male	Female		
**Left**
Anterior	1202.93 (4.21)	1180.06 (3.77)	14.92	**0.0001**
Middle	2458.53 (10.52)	2458.69 (9.43)	0.0001	0.99
Posterior	585.90 (4.07)	612.86 (3.64)	24.32	**0.000001**
**Right**
Anterior	1211.27 (5.22)	1185.55 (4.68)	13.44	**0.0003**
Middle	2346.08 (9.92)	2347.33 (8.89)	0.01	0.93
Posterior	564.98 (3.77)	580.66 (3.37)	9.59	**0.002**

*Note: The critical p value after Bonferroni correction was 0.008 (0.05/6 ROIs). Significant p values are in bold*.

**Figure 1:**
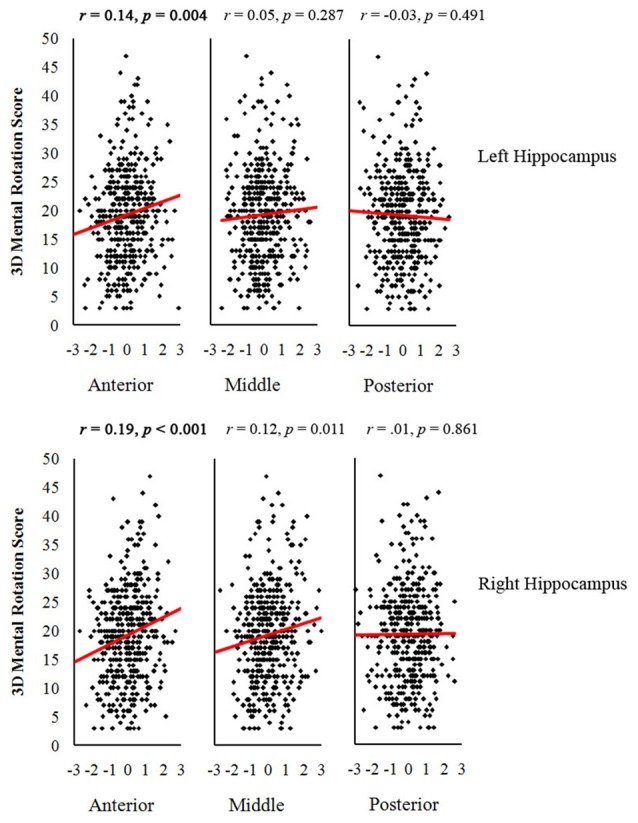
**Scatter plots between gray matter volumes (GMV) of anterior, middle and posterior hippocampus and 3-dimensional (3D) mental rotation performance (with the GMV of the whole brain as a covariate).** The critical *p* value after Bonferroni correction was 0.008 (0.05/6 ROIs). Significant *p* values are in bold.

Table 3 shows the correlations between GMVs of the different parts of the hippocampus and the scores of 3D mental rotation and the other two cognitive tasks after controlling for the whole brain GMV (see Supplementary Table 2S). The results showed that GMVs of the left and right anterior hippocampus were significantly correlated with performance on the 3D mental rotation task (left, *r* = 0.14, *p* < 0.008; right, *r* = 0.19, *p* < 0.008; see Figure 1 and Supplementary Figure 1S). After controlling for visual perception, the correlations between 3D mental rotation and the GMVs of the left and right anterior hippocampus remained significant (left, *r* = 0.14, *p* < 0.008; right, *r* = 0.19, *p* < 0.008). After controlling for intelligence, the correlation between 3D mental rotation and the GMVs of the right anterior hippocampus remained significant (right, *r* = 0.17, *p* < 0.008). After controlling for both visual perception and intelligence, the correlation between 3D mental rotation and the GMV of the right anterior hippocampus also remained significant (right, *r* = 0.18, *p* < 0.008). The correlations between mental rotation and the GMV of the left anterior hippocampus (*r* = 0.12, controlling for either intelligence alone or both intelligence and visual perception) were no longer significant after Bonferroni correction.

**Table 3:** **Correlation coefficients between gray matter volumes of the left and right hippocampus and behavioral performance (with the gray matter volume of the whole brain as a covariate)**.

	Left			Right		
	Anterior	Middle	Posterior	Anterior	Middle	Posterior
**3-D mental rotation**
*r*	**0.14**	0.05	−0.03	**0.19**	0.12	0.01
*p*	**0.004**	0.287	0.491	**0.000**	0.011	0.861
**MVTP**
*r*	0.08	0.10	0.01	0.07	0.07	0.04
*p*	0.119	0.045	0.881	0.156	0.135	0.43
**Raven’s advanced progressive matrices**
*r*	0.12	−0.00	0.02	0.11	0.00	0.08
*p*	0.015	0.989	0.629	0.028	0.943	0.087

*Note: The critical p value after Bonferroni correction was 0.008 (0.05/6 ROIs). Significant p values are in bold*.

ANCOVA was conducted to test whether the sex difference in 3D mental rotation could be explained by sex differences in GMVs of the hippocampus. After controlling for GMVs of the right anterior hippocampus and the whole brain, the sex difference in 3D mental rotation was no longer significant (*F*_(1,427)_ = 3.04, *p* = 0.070). The change in sex difference was statistically significant (*t* = 3.47, *p* < 0.01).

### Whole-Brain Analysis

**Figure 2:**
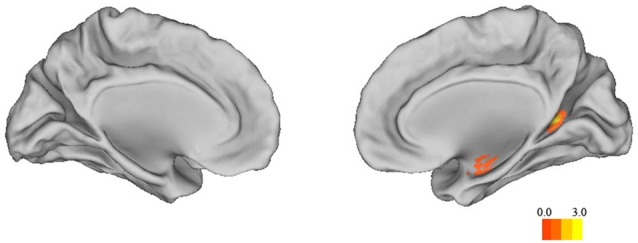
**The correlation map between 3D mental rotation performance and GMV of the whole brain (*p* < 0.05, FWE-corrected for multiple comparisons, cluster size >50)**.

To supplement the structural ROI analysis, we conducted a whole-brain analysis in SPM. Sex differences in the whole brain GMV are shown in Table 4. Correlation analysis confirmed that the structure of left and right anterior hippocampus was correlated with 3D mental rotation (*p* < 0.05, FWE-corrected for multiple comparison, peak, left: *x* = −34, *y* = −9, *z* = −19, *t* = 3.80; right: *x* = 35, *y* = −9, *z* = −17, *t* = 4.69; see Figure 2). In addition, GMV in the calcarine was significantly and positively associated with 3D mental rotation (*p* < 0.05, FWE-corrected for multiple comparison, cluster size >50; Table 5).

**Table 4:** **Loci showing significant sex differences in gray matter volume based on the whole brain analysis (*p* < 0.001, FWE-corrected for multiple comparisons, cluster size >200)**.

Brain region	Brodmann area	Cluster size	Coordinates			*t* value	*P*_corrected_
**Brain areas showing greater gray matter volumes in males than in females**
Right lingual	BA 17	21,486	12	−92	−13	13.06	<0.000001
	BA 17		53	−66	−22	12.43	<0.000001
	BA 17		43	−76	−17	11.49	<0.000001
	BA 30		9	−54	8	5.91	0.0004
Right hippocampus	BA 36	3467	12	−7	−34	9.09	<0.000001
	BA 36		19	−3	−31	8.88	<0.000001
Left precuneus	BA 30	2132	−4	−49	16	7.73	<0.000001
Left cerebellum		2041	−29	−73	−19	8.09	<0.000001
		1107	−30	−39	−56	10.3	<0.000001
		1013	−20	−59	−33	7.75	<0.000001
Left fusiform	BA 20	495	−30	−3	−44	6.69	0.000005
Right rectus	BA 47	453	20	13	−15	8.05	<0.000001
Right putamen		430	24	0	5	7.07	<0.000001
Right paracentral lobule	BA 5	416	4	−40	75	9.22	<0.000001
	BA 5		0	−27	76	7.96	<0.000001
	BA 5		−3	−44	72	6.68	0.000006
Right precuneus	BA 30		5	−47	14	7.69	<0.000001
Left amygdala		316	−23	−3	−25	6.37	0.00003
Left lingual	BA 18	209	−9	−84	−3	6.28	0.00005
	BA 18		−6	−74	0	5.98	0.0002
**Brain areas showing greater gray matter volumes in females than males**
Right thalamus		2381	3	−15	0	8.84	<0.000001
Left caudate		1709	−8	8	11	8.37	<0.000001
Right caudate		1284	8	6	9	7.72	0.00003
Right cerebellum		464	50	−75	−35	8.17	0.000002
Left cerebellum		381	−51	−72	−35	8.37	<0.000001

**Table 5:** **Loci showing positive correlations between 3-dimensional (3D) mental rotation and gray matter volumes based on the whole brain analysis (*p* < 0.05, FWE-corrected for multiple comparisons, cluster size >50)**.

Brain region	Brodmann area	Cluster size	Coordinates			*t* value	*P*_corrected_
Right calcarine	BA 30	397	23	−55	8	5.01	0.03
Right anterior hippocampus		393	35	−9	−17	4.69	0.03
	BA 28	66	24	4	−23	4.51	0.03

## Discussion

In the present study, we found that, compared to females, males performed better in 3D mental rotation and had greater GMV in the anterior hippocampus. The GMV of the right anterior hippocampus was significantly correlated with 3D mental rotation performance, even after controlling for visual perception and general intelligence. The sex difference in 3D mental rotation disappeared after controlling for GMV of the right anterior hippocampus.

### Sex Differences in Spatial Abilities

Our behavioral results were consistent with many previous studies that showed a stable male advantage in spatial ability, especially in 3D mental rotation. This male advantage may have had an evolutionary origin because of males’ role in hunting, for which a good sense of direction and a superior ability in spatial relations (throwing spears at the games) had been critical (Geary, 1995; Ecuyer-Dab and Robert, 2004). In modern-day life, males may no longer need to hunt, but they still prefer activities involving spatial processing such video games and sports (Okagaki and Frensch, 1994; Ozel et al., 2004; Robert and Héroux, 2004; Cherney and London, 2006), which may have continued to help males gain an advantage in spatial ability. Males and females have also been found to use different strategies when performing a navigation task, with males using spatial strategies and females using both verbal and spatial strategies (Merrill et al., 2016).

It is worth mentioning that, although Raven’s Progressive Matrices test also involves spatial processing and mental rotation, it did not show sex differences in our study. This result is consistent with previous studies (e.g., Raven, 1938; Eysenck and Kamin, 1981; Wei et al., 2012; Lütke and Lange-Küttner, 2015). There are at least two possible reasons. First, Raven’s Progressive Matrices aims to test general intelligence, so it includes measures of both visuospatial ability and reasoning. Using structural equation modeling, Schweizer et al. (2007) showed that the component of reasoning explained 46% of the variance of the total score, whereas mental rotation explained only 7% (Schweizer et al., 2007). Second, Raven’s mental rotation task involves only 2D rotation, which has smaller sex differences than 3D rotation (Peters et al., 1995; Roberts and Bell, 2003; Lütke and Lange-Küttner, 2015).

### Neural Basis of Sex Differences in Spatial Abilities

Sex difference in spatial processing has been associated with the volume and activation of the hippocampus. In nonhuman animals, the sex that is responsible for searching for food and nesting has a larger hippocampus (Clayton et al., 1997, and for a review see Lee et al., 1998). Our results showed that compared to females, males had a larger anterior hippocampus, which accounted for their advantage in 3D mental rotation. Consistently, previous research has linked a larger anterior hippocampus to better performance in encoding new spatial information (Maguire et al., 2006b). Our results on the role of the anterior hippocampus in mental rotation are consistent with the HIPER model (Lepage et al., 1998). According to this model, the anterior hippocampus is responsible for acquiring or encoding new visuospatial information, more specifically, coding information for head directions and angular features (Maguire et al., 1998), and registering new and abstract spatial environment (Save et al., 1992; Hartley et al., 2003; Sperling et al., 2003; Jackson and Schacter, 2004; Maguire et al., 2006b; Chua et al., 2007; Iaria et al., 2007; Doeller et al., 2008). Functional MRI studies also found that imagination tasks activated anterior hippocampus more than did memory tasks (Addis et al., 2007, 2009). The 3D mental rotation task seems to meet the requirements of this particular type of spatial information processing because it involves head directions and angular features and the 3D figures were likely to be new and abstract to the participants. Therefore, it seems likely that the anterior hippocampus subserves 3D mental rotation as tested in our project. Furthermore, it was the right anterior hippocampus that had a high correlation with 3D mental rotation after controlling for covariates. Consistent with such hemispheric specialization, previous fMRI studies have found that navigation tasks activate the right anterior hippocampus (Maguire et al., 1998; Mellet et al., 2000; Iaria et al., 2003, 2009; Doeller et al., 2008) and lesion studies have found that damages to the right anterior hippocampus impair spatial memory while damages to the left anterior hippocampus impair verbal memory (Abrahams et al., 1997; Bohbot et al., 1998). In sum, structural differences between males and females in the right anterior hippocampus seem to explain sex differences in 3D mental rotation.

Interestingly, the sex difference in the posterior hippocampus was opposite of that in the anterior hippocampus. Consequently, the whole hippocampus did not show sex difference in GMV. Some of the previous studies, however, have shown sex difference in the total GMV of the hippocampus (Giedd et al., 1997; Goldstein et al., 2001). One possible reason is age differences between studies. Goldstein et al. (2001) found that females in their 30 s (mean = 36.3 years of age) showed a larger hippocampus than their male counterparts and Pruessner et al. (2001) found that males in their 30 s began to show a decrease in their hippocampus. Our participants were all college students. Indeed previous studies found sex differences in developmental trajectories of the size of the hippocampus (Giedd et al., 1997; Pruessner et al., 2001; Suzuki et al., 2005).

It should be mentioned that, although our study focused on the hippocampus’s role in 3D mental rotation, our whole-brain analysis also found that the right calcarine was associated with 3D mental rotation. The calcarine sulcus is an important part of the primary visual cortex (V1; Sereno et al., 1995), which is important for all types of visual processing, but its specific role in 3D mental rotation needs further research. In addition to these two regions, previous studies found that mental rotation also elicited activation in other brain regions, but with mixed results on sex differences. One study found that males activated the frontal gyrus, cingulate cortex and occipital gyrus more than did females (Semrud-Clikeman et al., 2012), whereas another study found that females activated the middle temporal gyrus, frontal gyrus and primary motor cortex more than did males (Kucian et al., 2005). These cross-study differences in other brain regions may have been due to different levels of difficulty of the tasks (Roberts and Bell, 2003) or the particular paradigms used in different studies. Previous studies also found that the parietal lobe, rather than the hippocampus, had the highest correlation with spatial ability (Corbetta et al., 2000; Koscik et al., 2009; Hänggi et al., 2010). There are three possible explanations. First, the hippocampus is involved during the early brief stage of the spatial tasks (Iaria et al., 2003; Etchamendy et al., 2012), and thus its activation may be overshadowed by later stages of processing. Event-related potentials (ERP) studies also showed a late posterior negativity related to mental rotation tasks (Peronnet and Farah, 1989; Heil, 2002). Second, there are sex differences in neural bases of spatial processing, which might have complicated previous findings: males depend more on the hippocampus for spatial processing, whereas females depend more on the parietal and frontal lobes (Grön et al., 2000). Third, as mentioned earlier, task differences (using spatial or verbal strategies) affect the involvement of the hippocampus (Iaria et al., 2003; Bohbot et al., 2004).

Finally, although this study found significant sex differences in 3D mental rotation which could be accounted for by structural differences in the right anterior hippocampus, we should recognize that the brain is plastic and the structure of the hippocampus could be changed through training (Lövdén et al., 2010; Kühn and Gallinat, 2014; Kühn et al., 2014), even in the case of the aging brain (Kempermann et al., 1998; Maguire et al., 2000, 2006a; Lövdén et al., 2012). Training has been found to improve spatial ability (Sanchez, 2012; Sorby et al., 2013) and reduce or eliminate its sex difference (Terlecki and Newcombe, 2005; Feng et al., 2007; Spence et al., 2009; Tzuriel and Egozi, 2010). In addition, some researchers have also found that the GMV of females’ posterior hippocampus changes with menstrual cycle, showing increases from the early follicular phase to the late follicular phase (Lisofsky et al., 2015). Such an influence should be considered in future research of sex differences in the hippocampus, especially the posterior hippocampus.

## Author Contributions

CC, QD and XZ designed the study. WW analyzed the data. WW, CC and XZ wrote the manuscript.

## Conflict of Interest Statement

The authors declare that the research was conducted in the absence of any commercial or financial relationships that could be construed as a potential conflict of interest.
