# Supplementary material for: Sex Differences in Gray Matter Volume of the Right Anterior Hippocampus Explain Sex Differences in Three-Dimensional Mental Rotation
Source: Front Hum Neurosci. 2016 Nov 15;10:580. doi: 10.3389/fnhum.2016.00580 (PMC5108793; doi:10.3389/fnhum.2016.00580)

Supplementary Material:

In this supplementary material, we provide results from the data analysis that did not control for the gray matter volume (GMV) of the whole brain. The results were similar to those after controlling for the whole brain GMV as reported in the manuscript.

Structural ROI analysis

The GMV of the anterior hippocampus was larger for males than for females, whereas that of the posterior hippocampus was larger for females than males (see Table S1). There was no sex difference in GMV of the middle hippocampus.

The structural ROI analysis involved six brain areas (bilateral anterior, middle, and posterior hippocampus), so we used Bonferroni correction to adjust for the significance thresholds: $p = 0.05/6 = 0.008$. Table S2 shows the correlations between GMVs of the different parts of the hippocampus and the scores of the 3D mental rotation, visual perception, and intelligence tests. Results showed that GMV of the right anterior hippocampus was significantly correlated with performance on the 3D mental rotation task (see Figure 1S). After controlling for visual perception, the correlations between 3D mental rotation and GMV of the left and right anterior hippocampus remained significant (left, $r = 0.13$, $p < 0.008$; right, $r = 0.18$, $p < 0.008$). After controlling for intelligence, the correlations between 3D mental rotation and the gray matter volumes of the right anterior hippocampus remained significant (left, $r = 0.11$, *n.s.*; right, $r = 0.17$, $p < 0.008$). After controlling for visual perception and intelligence, the correlations between 3D mental rotation and the gray matter volumes of the right anterior hippocampus remained significant (left, $r = 0.12$, *n.s.*;

right, $r = 0.17$, $p < 0.008$).

ANCOVA was conducted to test whether the sex difference in 3D mental rotation could be explained by sex differences in GMVs of the hippocampus. After controlling for GMV of the right anterior hippocampus, the sex difference in 3D mental rotation was no longer significant [$F(1, 428) = 3.18$, $p = 0.075$].

Table 1S: Mean scores (and standard error) and sex differences in gray matter volumes (mm^3) of the left and right anterior, middle, and posterior hippocampus.

		Sex		*F*	*p*
		Male	Female		
Left	Anterior	1204.12 (4.34)	1180.11 (3.89)	16.97	**0.00005**
	Middle	2462.21 (11.03)	2455.74 (9.88)	.19	0.66
	Posterior	587.72 (4.38)	611.40 (3.93)	16.18	**0.00007**
Right	Anterior	1212.67 (5.36)	1184.43 (4.80)	15.39	**0.0003**
	Middle	2349.46 (10.37)	2344.61 (9.29)	.12	0.73
	Posterior	566.47(4.00)	579.45 (3.58)	5.84	**0.02**

Note: The critical *p* value after Bonferroni correction was .008 (0.05/6 ROIs). Significant *p* values are in bold.

Table 2S: Correlation coefficients between gray matter volumes of the left and right hippocampus and behavioral performance.

		Left			Right		
		Anterior	Middle	Posterior	Anterior	Middle	Posterior
3-D mental rotation	*r*	**0.13**	0.05	-0.04	**0.18**	0.11	0.00
	p	**0.007**	0.35	0.461	**0.000**	0.018	0.937
MVTP	*r*	0.05	0.06	-0.03	0.04	0.04	0.00
	p	0.324	0.197	0.569	0.38	0.407	0.931
Raven's Advanced Progressive Matrices	*r*	0.10	-0.02	0.00	0.09	-0.02	0.06
	p	0.045	0.678	0.984	0.071	0.745	0.239

Note: The critical *p* value after Bonferroni correction was 0.008 (0.05/6 ROIs). Significant *p* values are in bold.

Figure 1S: Scatter plots between gray matter volumes (mm3) of anterior, middle, and posterior hippocampus and three-dimensional mental rotation performance. The critical *p* value after Bonferroni correction was 0.008 (0.05/6 ROIs). Significant *p* values are in bold.

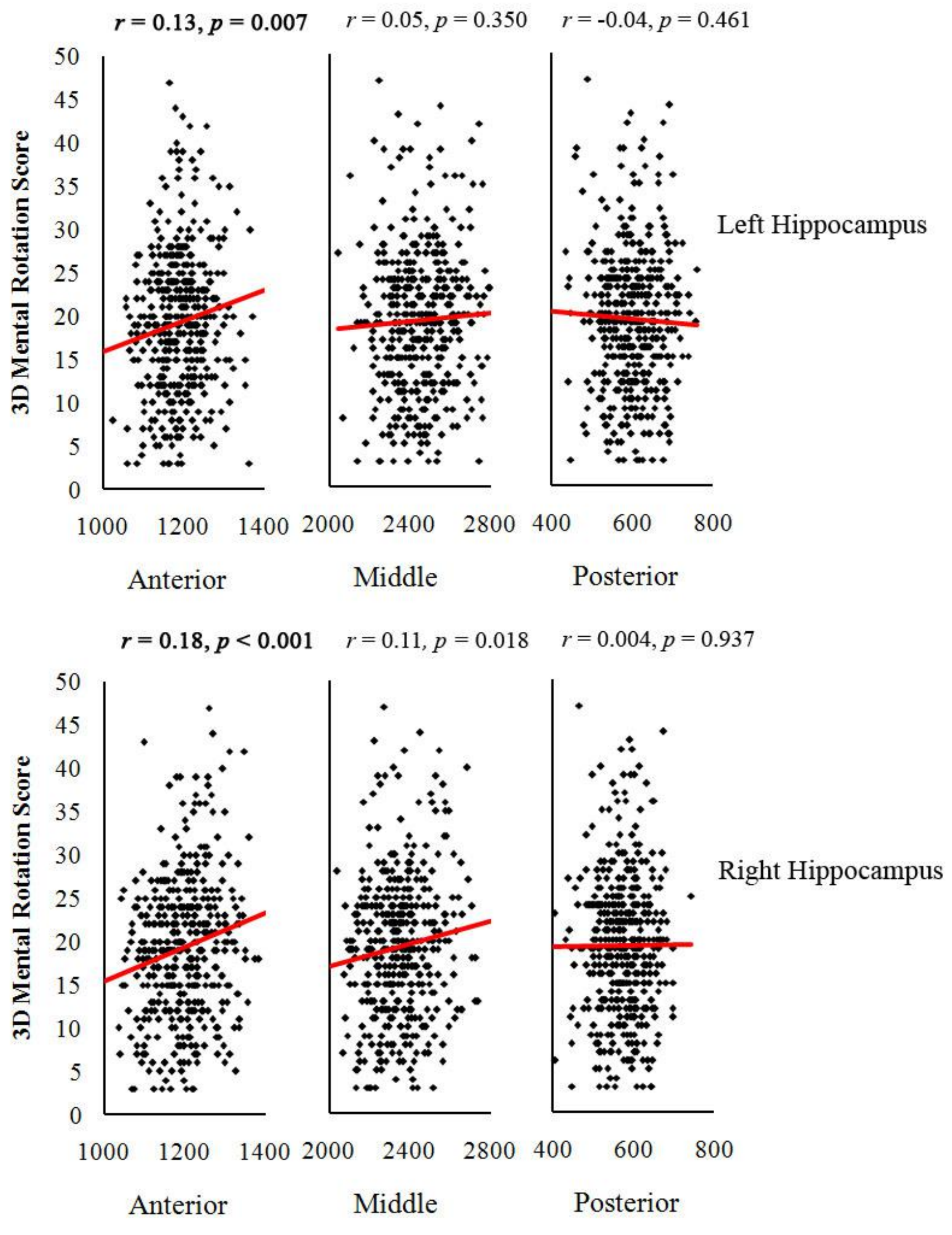

Supplement: Supplementary file 1 [file Presentation_1.pdf]
